# Traditional Chinese Medicine for Postoperative Care following Anterior Cruciate Ligament Reconstruction: A Systematic Review and Meta-Analysis

**DOI:** 10.1155/2021/9993651

**Published:** 2021-09-20

**Authors:** Hokyung Chang, Hyungsuk Kim, Koh-Woon Kim, Jae-Heung Cho, Mi-Yeon Song, Won-Seok Chung

**Affiliations:** ^1^Department of Rehabilitation Medicine of Korean Medicine, Kyung Hee University Medical Center, Seoul 02447, Republic of Korea; ^2^Department of Clinical Korean Medicine, Graduate School, Kyung Hee University, Seoul 02447, Republic of Korea

## Abstract

**Objectives:**

This review verifies the clinical effects of traditional Chinese medicine (TCM) combined with conventional rehabilitation after anterior cruciate ligament reconstruction (ACLR).

**Methods:**

MEDLINE/PubMed, EMBASE, CENTRAL, JMAS, CNKI, and seven Korean databases were searched using predetermined strategies. The risk of bias was assessed using Cochrane Collaboration's tool and a meta-analysis was conducted accordingly.

**Results:**

Nineteen randomized controlled trials involving 1283 participants were included in this systematic review and meta-analysis. The TCM treatment group showed more significant improvements in pain (MD −0.74, 95% CI [−0.93, −0.54]; I2 = 89%), range of motion (ROM) (SMD 1.19, 95% CI [0.78, 1.59]; I2 = 78%), and knee swelling (SMD −1.72, 95% CI [−2.38, −1.07]; I2 = 76%). The Lysholm score of the TCM treatment group significantly improved (MD 5.62, 95% CI [3.93, 7.32]; I2 = 84%) relative to the control group. The IKDC subjective score (MD 3.40, 95% CI [−0.61, 7.41]; I2 = 97%) and the hospital for special surgery (HSS) score did not improve initially (MD 6.79, 95% CI [−1.27, 14.86]; I2 = 97%) but did so during the subgroup analysis. TCM showed a long-term effect on the IKDC subjective score (MD −0.51, 95% CI [−1.69, 0.67]; I2 = 30%). A longer treatment period of 12 weeks showed more improvement (MD 5.96, 95% CI [0.69, 11.22]; I2 95%).

**Conclusion:**

TCM can be used as an adjuvant therapy to conventional rehabilitation for relieving pain, improving ROM and oedema, and facilitating better function of the knee joint after ACLR. However, this recommendation should be cautiously applied in clinical practice owing to the low quality of the included studies.

## 1. Introduction

The anterior cruciate ligament (ACL), which is important for stabilizing the knee joint, is the most commonly injured ligament in athletes and trauma victims. The annual incidence of isolated ACL tears is 68.6 per 100,000 person-years [1]. Although the appropriate treatment for an ACL injury depends on its severity and the characteristics of the patient, ACL reconstruction (ACLR) is commonly performed. ACLR generally involves arthroscopy using a graft to replace the injured ACL with the patellar, hamstring, or quadriceps tendon.

Successful ACLR requires appropriate physical rehabilitation focusing on muscle strengthening and the enhancement of balance and proprioception of the knee joint to help patients recover their mobility [2]. However, several rehabilitation programs are often interrupted by pain, stiffness, and swelling of the knee joint after ACLR. To date, there is no consensus yet as to what is the most appropriate rehabilitation program for successful recovery after ACLR [3].

Traditional Chinese medicine (TCM) is characterized by a holistic approach to diagnosis, pathophysiology, and treatment based on basic theories, such as the Yin–yang and Qi theories. Major components of TCM include acupuncture, herbal medicine, and other physical therapy, such as massages. The effectiveness of East-West integrative medicine, including acupuncture and herbal medicine, for postoperative care after knee surgery has been continuously discussed [4, 5]. A recent meta-analysis suggested that acupuncture can relieve postoperative pain and reduce opioid consumption after total knee arthroplasty (TKA) [6]. According to another systematic review, electroacupuncture (EA) can offer pain relief after TKA [7]. Several studies have verified the effects of TCM on pain after knee surgery, including TKA, open reduction, and internal fixation (OR/IF) of the knee joint. However, there has been no separate systematic review of the effects of TCM during postoperative care of ACLR. In addition, current systematic reviews involve neuromuscular electrical stimulation (NMES), continuous passive motion therapy (CPM), cryotherapy, and homeopathic arnica therapy, instead of TCM [8]. Therefore, we conducted a systematic review focusing on the effects of TCM on pain after ACLR.

This review assesses the clinical effects of TCM combined with conventional rehabilitation used for postoperative care of ACLR compared with conventional rehabilitation alone. The main objective was to verify the effects of TCM on pain after ACLR. The secondary objective was to reveal the effects of TCM on the range of motion (ROM), the comprehensive evaluation, and the swelling of the knee joint after ACLR. The comprehensive evaluation involved the use of various scales to assess symptoms and function of the knee joint after ACLR.

## 2. Methods

The protocol of this systematic review has been registered with the Open Science Framework (osf.io/zy2w8), and it follows the Preferred Reporting Items for Systematic Reviews and Meta-Analysis (PRISMA) guidelines [9]. The protocol of the current review has been published elsewhere [10].

### 2.1. Criteria for Consideration of Studies for This Review

#### 2.1.1. Types of Studies

This review included only prospective randomized controlled trials (RCTs) on the effects of TCM after ACLR. Nonrandomized controlled trials, retrospective chart reviews, observational studies, and case studies were excluded. There was no language restriction for the studies.

#### 2.1.2. Types of Participants

Patients who were treated with TCM after ACLR were included, and there were no restrictions in age, sex, and the type of procedure or grafts used during reconstruction surgery. The studies involving patients who had undergone other surgeries of the knee joint or suffered from severe comorbidities and complications after surgery were excluded.

#### 2.1.3. Types of Interventions

This study defined various physiotherapy interventions, patient education, and pharmacological treatments as standards of “conventional rehabilitation (CR)” during postoperative care following ACLR in clinical practice. Pharmacological treatments included various analgesics administered orally or through intravenous injections. Physiotherapy included rehabilitation programs such as mobilization of the knee joint using CPM, physical exercise, and skin electrical stimulation treatment.

*Experimental Group Intervention.* For the experimental group, the intervention had to include TCM treatments. In this review, we defined TCM as interventions including either acupuncture or herbal medicine. The combinations of acupuncture or herbal medicine and other TCM treatments, such as moxibustion, fuming-washing therapy, fumigation, and massaging along the meridian, were permitted accordingly. The combination of TCM and conventional rehabilitation was also permitted if the same treatments were provided to the control group.

With respect to acupuncture, various types of needling, provided that they punctured the skin, as well as other stimulations of the needle, such as electric or heat, were also included in this study. Modalities, which did not involve penetration, including acupressure and laser acupuncture, were not considered acupuncture.

For herbal medicine, all the orally administered forms were included as part of the study. Combinations of two or more types of herbal medicine were also included accordingly. There were no restrictions on the composition, intake dosage or frequency, and application duration.

*Control Group Intervention.* The control groups were treated with conventional rehabilitation (i.e. physiotherapy, analgesics, patient education). They were subject to no other restrictions under the assumption that the same treatments were applied to the intervention group. If a study involved a control group of patients who were treated with TCM therapy, it was excluded because this review was designed to compare TCM and other modalities.

#### 2.1.4. Types of Outcome Measures

*Primary Outcomes.* The primary outcomes included all the indicators for evaluating pain (i.e. visual analogue scale (VAS) and numerical rating scale (NRS)). If the pain scores during rest and activity were presented, those related to activity were selected because physiotherapy after ACLR included several activities for promoting muscle strength and flexibility in clinical practice.

*Secondary Outcomes.* The secondary outcomes included the ROM of the knee joint and indicators for evaluating symptoms, function of the knee joint (i.e. Lysholm score, International Knee Documentation Committee 2000 subjective knee form (IKDC Subjective score), Hospital for Special Surgery (HSS) score), and swelling of the knee joint taken from the knee circumference, after ACLR.

### 2.2. Search Methods for the Identification of Studies

The Cochrane Central Register of Controlled Trials (CENTRAL), MEDLINE/PubMed, and EMBASE were searched for articles. One Chinese database (Chinese National Knowledge Infrastructure; CNKI), one Japanese database (Japan Medical Abstracts Society; JMAS), and seven Korean databases (Korean National Assembly Digital Library, Korean Association of Medical Journal Editors, Oriental Medicine Advanced Searching Integrated System, Korean Studies Information Service System, National Digital Science Library, Database Periodical Information Academic, and Korean Traditional Knowledge Portal) were systematically searched for studies published from their inceptions to June 2020 by two reviewers (H. C., H. K.). The search process was based on specific keywords from four broad concepts of interest: (1) “anterior cruciate ligament reconstruction,” (2) “acupuncture,” (3) “Chinese herbal medicine,” and (4) “randomized controlled trial.” The complete search strategy for the CENTRAL, MEDLINE/PubMed, EMBASE, and CNKI databases is presented in the Appendix.

To find relevant literature omitted from the search above, the references of these papers were screened by their titles and abstracts. The World Health Organization International Clinical Trials Registry Platform (ICTRP) was explored for unpublished trials. Furthermore, literature that could not be searched online (i.e. hard copy) were manually searched. We also contacted the researchers of ongoing studies to verify information when required.

### 2.3. Data Collection and Analysis

#### 2.3.1. Selection of Studies

Using predetermined strategies, two reviewers (H. C., H. K.) independently searched the aforementioned databases. For database articles, ambiguous literature, and manually searched hard copies, the reviewers performed primary screening by applying predetermined inclusion and exclusion criteria after reading the titles and the abstracts. The predetermined criteria were applied to the full-texts of studies to select the RCTs for our systematic review. When consensus on the selection process could not be reached, a third reviewer (W. C.) made the final decision about including or excluding ambiguous studies.

#### 2.3.2. Data Extraction and Management

The reviewers extracted information from each article through a full-text review of the finally selected articles. When the collected data were incomplete or unclear, the arbiter contacted the authors of the original articles to request additional data or further explanation. We obtained data on the demographics of the sample, onset of ACL injury, date from ACLR to initial TCM treatment, details of intervention in experimental groups, details of intervention in control groups, types of outcome measurements, evaluation time points after surgery, and adverse events. The reviewers made final decisions on any issues following consultation with an arbiter (W. C.) when consensus could not be reached.

#### 2.3.3. Risk of Bias Assessment

Two independent reviewers evaluated the risk of bias of the included studies using the Cochrane Collaboration tool (risk of bias, ROB) to assess the quality of each RCT. Seven domains were used for the assessment: random sequence generation, allocation concealment, blinding of participants and personnel, blinding of outcome assessment, incomplete outcome data, selective outcome reporting, and other sources of bias. Each domain was rated as high risk, low risk, or unclear risk. When a consensus on the assessment of the ROB could not be reached through consultations, a third reviewer (W. C.) was consulted to make a final decision.

#### 2.3.4. Quantitative Data Synthesis

The mean differences (MDs) with 95% confidence intervals (CIs) were used for the analysis of continuous data. Weighted mean differences (WMDs) were adopted when the same scale was used, whereas standardized mean differences (SMDs) were used if different indicators were used to measure certain outcomes of the included studies.

When a study reported multiple group comparisons, only data from the treatment group that received more intensive conventional intervention were included in the analysis. For studies with a crossover research design, data from the first sessions were obtained accordingly.

We conducted a meta-analysis to estimate the differences between groups in the primary and secondary outcomes using the Cochrane Collaboration software (Review Manager Software Version 5.3). Depending on the level of heterogeneity among the included studies, we applied a fixed-effects model or a random-effects model. When heterogeneity was relatively high (I^2^ > 50%), a random-effects model with 95% CI was used to analyze the pooled effect estimates [11]. Heterogeneity was assessed in the following three ways according to the guidelines of the Cochrane Handbook for Systematic Reviews of Interventions: (1) a visual check of the forest plot, (2) using a heterogeneity *χ*^2^ test, and (3) using Higgins I^2^ statistic. In interpreting the heterogeneity *χ*^2^ test, a significance level of *p* < 0.10 was used to represent meaningful heterogeneity. A value of Cochrane's Higgins I^2^ greater than 75% represented considerable heterogeneity. A subgroup analysis was conducted to identify the reasons for heterogeneity when considerable heterogeneity was detected. If meaningful heterogeneity could not be explained by subgroup analysis, we did not conduct a meta-analysis.

#### 2.3.5. Subgroup Analysis

When it was necessary to explain the considerable heterogeneity of the included studies, we conducted a subgroup analysis based on the following: (1) the type of TCM treatments (i.e. acupuncture alone, herbal medicine alone, acupuncture plus herbal medicine, acupuncture plus more than one other TCM treatment, herbal medicine plus more than one other TCM treatment, acupuncture plus herbal medicine with more than one other TCM treatment), (2) the time points of evaluation after reconstruction surgery (<2 weeks, 2–4 weeks, 4–8 weeks, 8–12 weeks, 12–16 weeks, 16 weeks–1 year, and more than a year), and (3) duration of treatment (<2 weeks, 2–4 weeks, 4–7 weeks, 7–8 weeks, more than 12 weeks). The time points of evaluation were established according to the stage of rehabilitation after ACLR [12] because symptoms such as pain, ROM, oedema, and dysfunction of the knee joint differ with the stage of rehabilitation.

## 3. Results

### 3.1. Included Studies

**Figure 1:**
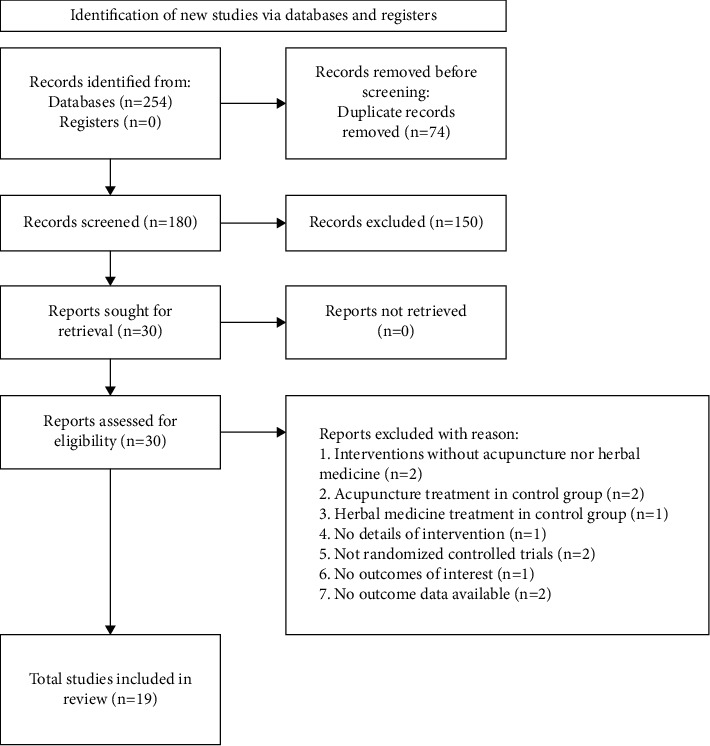
Flow diagram of the study selection process.

A total of 254 articles were retrieved from the online search. Initially, two of the authors (H.C., H.K.) screened the articles, and 74 records were removed because of duplicates. The title and abstracts of the remaining articles were further examined for eligibility, and 151 records were eliminated for several reasons: not being about ACLR, interventions without acupuncture nor herbal medicine, acupuncture treatment in the control group, no outcomes of interest, not RCTs, animal studies, ongoing research without available results, and inaccessible full texts. Subsequently, the full-text articles were assessed for eligibility, and 10 records were eliminated for various reasons: interventions without acupuncture nor herbal medicine, acupuncture treatment in the control group, herbal medicine treatment in the control group, not RCTs, no outcome of interest, and insufficient outcome data which were not obtained even though we had contacted the author(s) of the original study. Finally, 235 articles were excluded, and 19 RCTs involving a total of 1283 patients were included for analysis. The reasons for exclusion and the selection flow are presented in Figure 1.

### 3.2. Characteristics of the Studies

The characteristics of the included studies are presented in Table 1. The included RCTs were published between 2006 and 2020, with 18 of them published in Chinese [13–30] and one of them published in English [31]. Of the included studies, 18 were implemented in China [13–30] and one in Spain [31]. The sample sizes of the studies ranged from 25 to 160. All the patients in the studies had undergone ACLR for ACL injury, and their mean ages were between 25 and 38. The onset of ACL injury varied from 4 hours to 3 years, and the time of initiation of intervention after reconstruction surgery ranged from immediately after surgery to approximately 4 months after surgery.

Various TCM treatments were applied to the experimental group. Seven studies adopted only acupuncture as TCM treatment, including manual acupuncture alone [15, 22, 30, 31], manual acupuncture combined with warm needling [23], or manual acupuncture combined with electroacupuncture [16, 21]. One study used manual acupuncture and electroacupuncture combined with fuming-washing therapy and massaging along the meridian [14]. In three studies, patients were treated with herbal medicine alone [20, 28, 29], whereas in the other three, patients were treated with herbal medicine combined with fuming-washing therapy [24] or massaging along the meridian [17, 18]. In five studies, acupuncture and herbal medicine were adopted as the main TCM treatments. Of them, three used electroacupuncture and herbal medicine combined with massaging along the meridian [19] or fuming-washing therapy [25, 26], while one study used both manual acupuncture and electroacupuncture combined with herbal medicine and fuming-washing therapy [13]. The other one used manual acupuncture and herbal medicine combined with fuming-washing therapy and massaging along the meridian [27].

The durations of the treatments in the experimental groups ranged from a day [31] to one year [20]. The details of the acupuncture treatments are presented in Table 2. Acupuncture was performed from the western medical point of view targeting the vastus medialis muscle trigger point (TrP) in only one study [31], whereas the others used specific acupuncture points near the affected knee joint based on the TCM theory. The top ten frequently used acupuncture points were SP10, ST36, GB34, SP9, SP6, ST34, BL57, ST32, ST35, and BL39, all of which were near the muscles of the affected knee joints. The frequently used acupuncture points are organized in Table 3. Acupuncture treatments were applied for durations between 15 minutes and 30 minutes accordingly.

The dosage forms and frequencies of the herbal medicine treatments varied with each study, and the details are presented in Table 4. The durations of the herbal medicine treatments ranged from three weeks [13] to one year [20]. The most frequently used Chinese medicinal herb was Angelicae Gigantis Radix. Two studies [25, 27] did not provide any information about the basic components of herbal medicine. The frequently used Chinese medicinal herbs are organized in Table 5.

All the patients in the control groups had undergone conventional rehabilitation, including physiotherapy, patient education, and pharmacological treatments. The same treatments were applied to the experimental group.

Fifteen studies used the VAS as the main indicator for measuring pain after ACLR [13–19, 21–26, 31]. One study did not present standard deviation data and was thus excluded from the analysis because we could not receive any data from the author of the original study [17]. In three studies [19, 25, 26], the VAS scores obtained during rest and activity were presented, and the VAS score during activity was included in the analysis as planned. Ten studies presented the ROM of the affected knee joint. Four studies [14, 18, 23, 26] measured the active ROM, and one study [15] measured the passive ROM of the affected knee joint. The other four studies [19, 24, 25, 31] did not mention specific measurement methods Fourteen studies [13, 14, 17–20, 22–29] reported the Lysholm scores, six studies [17, 18, 20, 25, 26, 28] reported the IKDC subjective scores, and three studies [21, 25, 30] presented the HSS scores, which are comprehensive indicators for evaluating the symptoms and function of the knee joint after ACLR. Four studies [14, 15, 18, 24] measured the circumferences of the affected knee joint near the patella bone to evaluate knee joint swelling.

Several studies reported adverse events after TCM treatment. One study [31] reported that three patients suffered hemorrhages larger than 4 cm^2^, which were not significantly different in the experimental and control groups (*p*=0.073). Another study [25] reported that three patients suffered from diarrhea, which resolved spontaneously after taking herbal medicine. One study [16] compared adverse events such as headache, nausea, vomiting, abdominal pain, diarrhea, leg oedema, and dizziness in the electroacupuncture and the analgesics groups. Of 20 patients in the control group, three complained of nausea, vomiting, and abdominal pain after taking analgesics, whereas only one patient of 20 in the experimental group complained of dizziness after acupuncture treatment. The difference was statistically significant (*p*=0.004).

### 3.3. Risk of Bias in Included Studies

**Figure 2:**
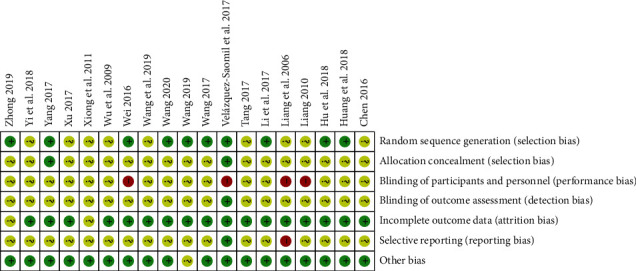
Assessment of the risk of bias.

Of the studies included in this review, 10 [14–16, 20, 22–24, 28, 30, 31] used the appropriate randomization method, while nine studies [13, 17–19, 21, 25–27, 29] did not provide specific descriptions about their randomization methods (Figure 2). Two studies [20, 31] adequately carried out allocation concealment using opaque closed-letter envelopes, while the other studies did not describe their approaches to allocation concealment in detail. No studies were evaluated as low-risk based on participant and personnel blinding because it was difficult to exclude performance bias in administering the TCM treatments. Fifteen studies [13–15, 17–24, 27–30], which involved single-blinded participants, were evaluated as high risk. The other studies did not indicate participant blinding. Only one study [31] provided clarifications on the blinding methodologies and assessment of outcomes. The other studies did not clarify the specific methods used to prevent detection bias. Three studies [20, 28, 31] showed dropouts within the domain of incomplete outcome data, but this was considered insignificant because the dropout rates of the groups were very low and similar between experimental groups and control groups, respectively. The other two studies [18, 22] did not indicate dropout or withdrawal. In seventeen studies, selective reporting was not decided and thus reporting was ambiguous. One study [31] published its protocol, and all of the prespecified outcomes were reported in the results. Another study [26] reported different evaluation time points in the methods and the results sections. Other forms of bias were evaluated based on whether there was no significant difference between the general characteristics of the patients in the experimental and control groups before treatment. All the included studies, except one [28], clarified that there was no significant difference between the general characteristics of the groups.

### 3.4. Effects of Interventions

Nineteen studies [13–31] involving a total of 1283 participants were included in the meta-analysis. The meta-analysis was based on six outcomes: the VAS score for pain evaluation, ROM for measuring joint mobility, Lysholm score, IKDC subjective score, HSS score for the comprehensive evaluation of the symptoms and function of the knee joint, and knee circumference for measuring knee swelling. Because the methods for measuring ROM and the knee circumference were different in each study, SMD was employed for the meta-analysis. A subgroup analysis was conducted for all the outcomes because considerable heterogeneities were revealed in the analysis of the pooled effect.

#### 3.4.1. Primary Outcomes

**Figure 3:**
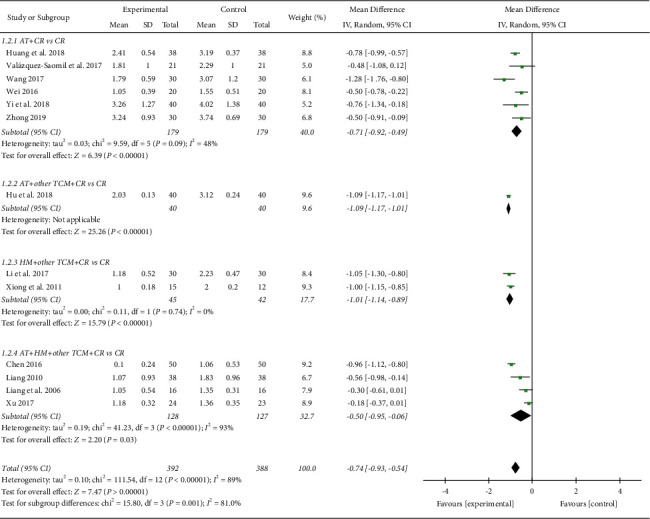
VAS score (subgroup analyzed by treatment types). AT: acupuncture treatment; CR: conventional rehabilitation; TCM: traditional Chinese medicine; HM: herbal medicine.

**Figure 4:**
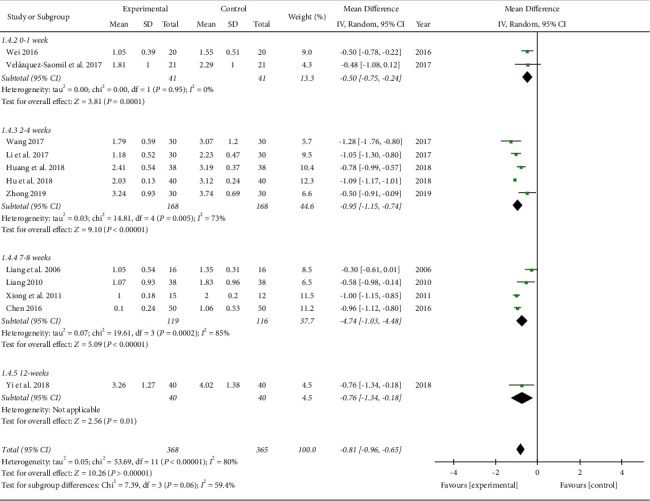
VAS score (subgroup analyzed by treatment duration).

In the meta-analysis of thirteen studies [13–16, 18, 19, 21–26, 31] involving 780 subjects, the TCM group showed more significant improvement in the VAS score after TCM treatment than the control group (MD −0.74, 95% CI [−0.93, −0.54]; I2 = 89%; Figure 3). As planned, the subgroup analysis was based on the types of TCM treatments, evaluation time points after surgery, and durations of treatment. All the TCM treatment types, including the acupuncture (MD −0.71, 95% CI −0.92 to −0.49, and I2 48%), acupuncture plus other TCM (MD −1.09, 95% CI [−1.17, −1.01]), herbal medicine plus other TCM (MD −1.01, 95% CI [−1.14, −0.89]; I2 = 0%), and acupuncture plus herbal medicine combined with other TCM (MD −0.50, 95% CI [−0.95, −0.06]; I2 = 93%) showed significantly better pain relief after surgery than the control group (Figure 3). For the evaluation time points after surgery, all the periods between 0-2 weeks after surgery (MD −0.50, 95% CI [−0.73, −0.27]; I2 = 0%), 2–4 weeks after surgery (MD −1.28, 95% CI [−1.76, −0.80]), 4–8 weeks after surgery (MD -1.00, 95% CI [−1.18 to −0.82]; I2 = 40%), 12–16 weeks after surgery (MD −0.62, 95% CI [−1.04, −0.20]; I2 = 95%), and more than 16 weeks after surgery (MD −0.74, 95% CI [−0.92, −0.54]; I2 = 0%) were associated with significantly better pain relief than the control group (not shown). For treatment duration, TCM was more effective for pain relief than the control regardless of whether the treatment duration was short or long (Figure 4): 0–1 week (MD −0.50, 95% CI [−0.75, −0.24]; I2 = 0%), 2–4 weeks (MD −0.95, 95% CI [−1.15, −0.74]; I2 = 73%), 7–8 weeks (MD −0.74, 95% CI [−1.03, −0.46]; I2 = 85%), 12 weeks (MD −0.76, 95% CI [−1.34, −0.18]).

#### 3.4.2. Secondary Outcomes

**Figure 5:**
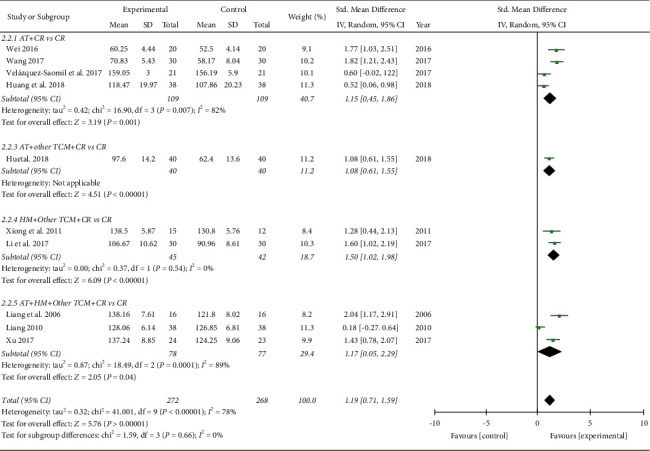
ROM (subgroup analyzed by treatment types). AT: acupuncture treatment; HM: herbal medicine; TCM: traditional Chinese medicine; CR: conventional rehabilitation.

*ROM of the Knee Joint.* In the meta-analysis of 10 studies involving 540 patients, the TCM group showed significantly better improvement in the knee ROM than the control group (SMD 1.19, 95% CI [0.78, 1.59]; I2 = 78%; Figure 5). In the subgroup analysis, all the TCM treatments were more effective than the control for ROM improvement regardless of the TCM treatment types: acupuncture (SMD 1.15, 95% CI [0.45, 1.86]; I2 = 82%), acupuncture plus other TCM (SMD 1.08, 95% CI [0.61, 1.55]), herbal medicine plus other TCM (SMD 1.50, 95% CI [1.02, 1.98]; I2 = 0%), and acupuncture plus herbal medicine combined with other TCM (SMD 1.17, 95% CI [0.05, 2.29]; I2 = 89%). The TCM-related improvements in ROM after ACLR were not only short-term but also long-term relative to the controls (not shown): 0–2 weeks (SMD 1.77, 95% CI [1.03, 2.51]), 2–4 weeks (SMD 1.82, 95% CI [1.21, 2.43]), 4–8 weeks (SMD 1.10, 95% CI [0.58, 1.63]; I2 = 62%), 12–16 weeks (SMD 1.54, 95% CI [1.10, 1.99]; I2 = 0%), and more than 16 weeks (SMD 0.35, 95% CI [0.02, 0.68]; I2 = 6%).

#### 3.4.3. Comprehensive Outcomes of the Knee Joints

**Figure 6:**
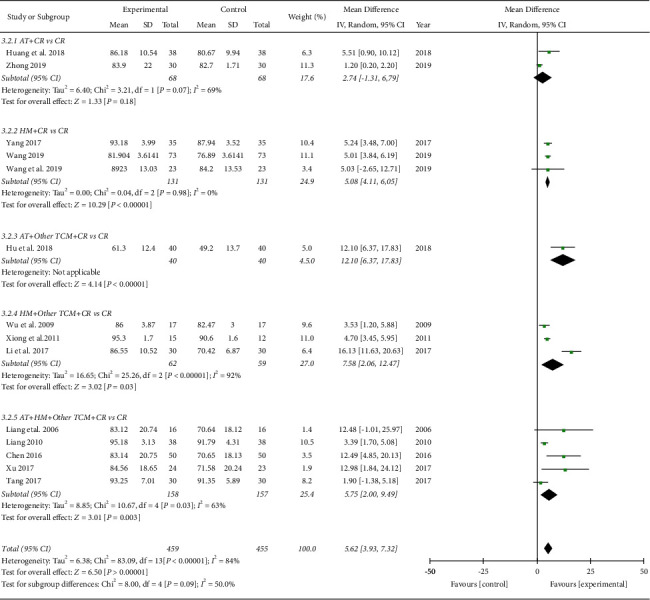
Lysholm score (subgroup analyzed by treatment types). AT: acupuncture treatment; HM: herbal medicine; TCM: traditional Chinese medicine; CR: conventional rehabilitation.

*Lysholm Score.* In the meta-analysis of fourteen studies involving 914 subjects, the TCM group showed significantly better improvement in the Lysholm score than the control group (MD 5.62, 95% CI [3.93, 7.32]; I2 84%; Figure 6). All types of TCM treatments showed greater improvement in the Lysholm score except for one study using acupuncture alone: acupuncture (MD 2.74, 95% CI [−1.31, 6.79]; I2 = 69%), herbal medicine (MD 5.08, 95% CI [4.11, 6.05]; I2 = 0%), acupuncture plus other TCM (MD 12.10, 95% CI [6.37, 17.83 ]), herbal medicine plus other TCM (MD 7.56, 95% CI [2.66, 12.47]; I2 = 92%), and acupuncture plus herbal medicine combined with other TCM (MD 5.75, 95% CI [2.00, 9.49]; I2 = 63%). Regardless of the time points of evaluation after surgery, the TCM group showed a significantly better improvement in the Lysholm score than the control group (not shown): 4–8 weeks (MD 11.75, 95% CI [5.85, 17.64]; I2 = 67%), 12–16 weeks (MD 4.75, 95% CI [2.06, 7.43]; I2 = 84%), 16 weeks-1 year (MD 3.64, 95% CI [2.05, 5.23]; I2 = 0%), and 1 year (MD 4.64, 95% CI [3.26, 6.02]; I2 = 41%). The TCM group showed better improvement in the Lysholm score than the control group, regardless of the treatment duration (not shown): 4 weeks (MD 7.90, 95% CI [1.33, 14.48]; I2 = 93%), 7-8 weeks (MD 4.62, 95% CI [3.33, 5.91]; I2 = 46%), 12 weeks (MD 4.64, 95% CI [3.26, 6.02]; I2 = 41%).

**Figure 7:**
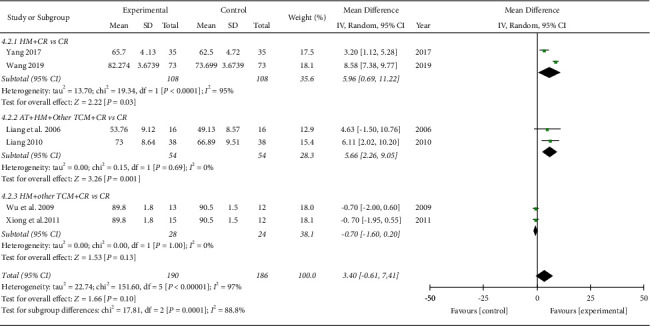
IKDC subjective score (subgroup analyzed by treatment types). AT: acupuncture treatment; HM: herbal medicine; TCM: traditional Chinese medicine; CR: conventional rehabilitation.

**Figure 8:**
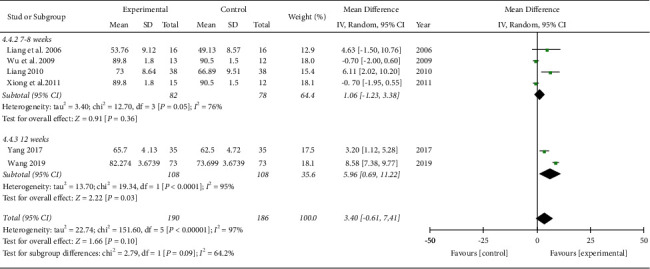
IKDC subjective score (subgroup analyzed by treatment duration).

*IKDC Subjective Score.* A meta-analysis of six studies involving 376 subjects showed that there was no significant difference between the IKDC subjective scores of the TCM and control groups based on the analysis of the pooled effect (MD 3.40, 95% CI [−0.61, 7.41]; I2 = 97%; Figure 7). In two RCTs [17, 18], the TCM group showed no significant improvement in the IKDC subjective score. In the subgroup analysis based on the TCM treatments, the herbal medicine group (MD 5.96, 95% CI [0.69, 11.22]; I2 = 95%) and acupuncture plus herbal medicine combined with the other TCM group (MD 5.66, 95% CI [2.26, 9.05]; I2 = 0%) showed significant improvements in the IKDC subjective score, whereas the herbal medicine combined with the other TCM group showed no significant improvement in the IKDC subjective scores (MD −0.70, 95% CI [−1.60, 0.20]; I2 = 0%; Figure 7). For the evaluation time points after surgery, TCM showed long-term improvement in the IKDC subjective score: 12–16 weeks (MD −0.51, 95% CI [−1.69, 0.67; I2 = 30%), 16 weeks-1 year (MD 6.11, 95% CI [2.02, 10.20]), and 1 year (MD 5.96, 95% CI [0.69, 11.22]; I2 = 95%; not shown). In addition, a longer duration of treatment was associated with significant improvement in the IKDC subjective score: 7-8 weeks (MD 1.06, 95% CI [−1.23, 3.36]; I2 = 76%) and 12 weeks (MD 5.96, 95% CI [0.69, 11.22]; I2 = 95%; Figure 8).

*HSS Score.* In the meta-analysis of three studies involving 316 subjects, the TCM group showed no significant improvement in the HSS score compared with the control group based on the pooled effect (MD 6.79, 95% CI [−1.27, 14.86]; I2 = 97%; not shown). In the subgroup analysis, the TCM groups showed significantly better improvements in the HSS score than the control groups regardless of the treatment method (not shown): acupuncture group (MD 9.41, 95% CI [1.62, 17.20]; I2 = 87%) and acupuncture plus herbal medicine combined with the other TCM group (MD 2.15, 95% CI [0.38, 3.92]). The TCM groups also showed significant improvements in the HSS score compared with the control groups regardless of treatment duration (not shown): 0–2 weeks (MD 13.03, 95% CI [10.90, 15.16]), and more than 2 weeks (MD 2.52, 95% CI [0.63, 4.41], and I2 = 6%).

*Knee Circumference.* In the meta-analysis of four studies involving 227 subjects, the TCM groups showed significant reductions in knee circumference compared with the control groups (SMD −1.72, 95% CI [−2.38, −1.07]; I2 = 76%; not shown). In the subgroup analysis based on the TCM treatments, all the subgroups showed significant reductions in the knee circumference than the control groups (not shown): acupuncture (SMD −2.65, 95% CI [−3.35, −1.94]), acupuncture plus other TCM (SMD −1.90, 95% CI [−2.43, −1.37]), and herbal medicine plus other TCM (SMD −1.21, 95% CI [−1.81, −0.60]; I2 = 37%). The TCM groups showed significant reductions in the knee circumference regardless of the evaluation time points after surgery (not shown): 2–4 weeks (SMD −2.65, 95% CI [−3.35, −1.94]), 4–8 weeks (SMD −1.69, 95% CI [−2.12, −1.26]; I2 = 18%), and more than 8 weeks (SMD −0.83, 95% CI [−1.63, −0.03]). Treatment durations of 2–4 weeks (SMD −1.97, 95% CI [−2.60, −1.35]; I2 = 70%) and 7 weeks (SMD −0.83, 95% CI [−1.63, −0.03]) were also associated with significant reductions in the knee circumference (not shown).

## 4. Discussion

Appropriate postoperative care is important for satisfactory outcomes after ACLR. The medical team should always be aware of signals such as knee pain, joint stiffness, and knee oedema because planning for individual rehabilitation is based on this information [32, 33]. Current rehabilitation programs usually focus on pain control, achieving a normal ROM and reducing joint effusion [34]. A small reduction (3°–5°) in the knee ROM after ACLR results in weaker quadriceps and increased risks of postoperative complications such as arthrofibrosis [35]. During the early postoperative phase, ambulation without pain indicates whether patients will be able to walk without crutches. During the late postoperative phase, knee joint effusion is a milestone of prognosis after ACLR [36]. The Lysholm score, IKDC subjective score, and HSS score were developed for evaluating the status of the knee joint after a ligament injury. Items for assessing the symptoms and function of the knee joint after knee surgery are included in these scales, which are commonly used for evaluating prognosis after ACLR [37, 38]. For example, the decision to return to normal sporting activities is based on IKDC subjective scores of more than 70 points [12]. Therefore, we selected the VAS score as the primary outcome, while ROM, knee circumference, Lysholm score, IKDC subjective score, and HSS score were chosen as the secondary outcomes in this review.

Currently, there are several rehabilitation protocols for postoperative care after ACLR. However, the most appropriate rehabilitation modality is still being debated on [2, 3]. A consensus cannot be reached because the postoperative conditions of patients differ in each case. Postoperative conditions are affected by various symptoms and the function of the knee joint, which may interrupt scheduled rehabilitation programs. Therefore, there is an increasing demand for updated rehabilitation programs for relieving symptoms and promoting the function of the knee joint after ACLR [4].

For these reasons, TCM may be used as adjuvant therapy for rehabilitation after ACLR because it is a more active intervention in that it can directly stimulate the muscles around the knee joint and promote systemic recovery compared to conventional rehabilitation. A recent systematic review revealed that the combination of TCM and CPM can promote better recovery of the knee-joint function after knee surgeries, including ACLR, TKA, and OR/IF versus CPM alone [39]. Therefore, we conducted a systematic review and meta-analysis to verify the clinical effects of TCM combined with conventional rehabilitation during postoperative care after ACLR.

This meta-analysis found that TCM combined with conventional rehabilitation can improve postoperative pain after ACLR. The VAS score, which was the primary outcome of this review, significantly decreased in the TCM groups regardless of the TCM treatment type, evaluation time points, and treatment duration. The knee ROM also significantly increased regardless of TCM intervention or evaluation time points after surgery. This meta-analysis also revealed that TCM treatments can significantly reduce knee swelling after ACLR regardless of their types, evaluation time points, and treatment duration. These results may be attributed to the analgesic effect of acupuncture treatment and the anti-inflammatory effect of herbal medicine.

The most frequently used acupuncture point was SP10, which is located in the belly of the vastus medialis muscle. Moreover, 10 most frequently used acupuncture points were all located within the affected lower limb, especially at the quadriceps femoris, tibialis anterior, and the gastrocnemius muscle. Unlike this review, a recent systematic review [6] and retrospective study [40] dealt mostly with trials in which acupuncture treatments were applied on the distal or contralateral part of the operated limb after TKA. While those studies validated the analgesic effect of acupuncture that lasted for a short-term only, this meta-analysis proved that the analgesic effect of localized acupuncture treatment in affected knees lasted for a up to one year. According to an RCT comparing the effect of using local acupoints and distal acupoints in degenerative knee osteoarthritis, localized acupuncture was more effective in improving the Western Ontario and McMaster Universities Osteoarthritis Index (WOMAC) score by directly stimulating the structures around the knee joint [41]. The localized analgesic effect of acupuncture can be achieved through adenosine A1 receptor mediation and axonal reflex, which stimulate the secretion of neuropeptides such as calcitonin gene-related peptide (CGRP), adenosine, and nitric oxide (NO). These substances dilate blood vessels and promote blood circulation [42, 43]. Acupuncture also has a segmental effect mediated by the gate control theory in addition to its regulation of the descending inhibitory system at the subcortical and cortical levels [44]. Moreover, the restriction of ROM after surgery is often caused by the stiffness of muscles around the knee joint [34, 45]. Therefore, acupuncture treatment at these muscles may improve the ROM of the knee joint by promoting blood flow around the knee joint [46].

Herbal medicine is often used for knee osteoarthritis and its analgesic effect is proven in several systematic reviews [47, 48]. Herbal medicine can also reduce the incidence rates of deep vein thrombosis after lower extremity orthopedic surgery [49]. Angelicae Gigantis Radix, which was used most frequently in the RCTs, is known to demonstrate an anti-inflammatory effect by inhibiting proinflammatory mediators such as the tumor necrosis factor-alpha (TNF-*α*), interleukin-6 (IL-6), IL-10, and vascular endothelial growth factor (VEGF) in lipopolysaccharide (LPS)-stimulated mouse macrophages [50]. The second most used components in herbal medicine were Paeoniae Radix, Achyranthis Radix, and Chaenomelis Fructus. Monoterpenoids in Paeoniae Radix serve as potential leads for the development of anti-inflammatory agents [51]. Achyranthis Radix usually serves as a lower-guiding drug and also enhances the therapeutic effect of TCM on the lower limbs and improves the supply of blood in the inflamed joint [52]. Moreover, quercetin in Chaenomelis Fructus can be a potent source for anti-inflammatory agents [53], and recent studies have shown that its components have anti-inflammatory properties which were effective for arthritis in a rat model [54].

Among the scales used for the comprehensive evaluation of the knee joint after ACLR, only the Lysholm score significantly improved in the TCM group based on the analysis of the pooled effect. Owing to the considerable heterogeneity involved, we conducted a subgroup analysis. During the subgroup analysis of Lysholm scores and IKDC subjective scores based on the TCM treatment types, the acupuncture groups and the herbal medicine plus other TCM treatment groups showed no improvement in Lysholm scores and IKDC subjective scores, respectively. However, the TCM groups, including both acupuncture and herbal medicine, showed significant improvements in Lysholm scores and IKDC subjective scores with low heterogeneity, which suggests that more diverse TCM treatments tend to be effective for relieving symptoms and promoting the function of the knee joint after ACLR. In the subgroup analysis of the IKDC subjective score based on the evaluation time points, TCM had begun to show effects in the past 16 weeks at the earliest with a relatively long treatment duration of 12 weeks. A report of a clinically important difference in the IKDC subjective score after 11.5–20.5 (range 6–28) months in patients who underwent surgical procedures of the knee joint may explain this [55]. In the subgroup analysis of the HSS score based on the treatment duration, TCM was associated with significant improvements in all the subgroups with low heterogeneity.

Three trials reported adverse events, including hemorrhage or dizziness after acupuncture and diarrhea after taking herbal medicine. However, these cases were mild, and the symptoms resolved spontaneously without life-threatening complications. In one study [16], acupuncture was associated with significantly fewer adverse events than taking analgesics. Some systematic reviews reported cases of local infection after acupuncture treatment [56, 57]. However, in this review, although acupuncture points used in the included RCTs were usually near the affected knee joint, serious adverse events such as surgical site infection did not occur. Therefore, this systematic review showed that TCM treatments for patients after ACLR were comparatively safe. However, further studies are needed to validate our findings because only a few of the included RCTs reported adverse events.

All the included studies were RCTs. Among the seven domains in ROB, performance bias was mainly assessed because it can affect subjective outcomes such as the VAS score, Lysholm score, IKDC subjective score, and HSS score [58]. The experimental groups were treated with TCM treatments and conventional rehabilitation, whereas the control groups were treated with conventional rehabilitation alone; therefore, it seemed impossible to blind the participants completely from the TCM treatments. Accordingly, performance bias was considered not clear or relatively high in all the included studies, and the quality of evidence was downgraded by one level for the ROB of the subjective outcomes. The quality of evidence was also downgraded by one for the inconsistency of the results because of the considerable heterogeneity although it changed during the subgroup analysis. There was no evidence of indirectness because all the studies directly compared the interventions. Most studies used adequate population sizes. However, in relation to the IKDC subjective score, HSS score, and knee circumference, the population size was relatively low, and the quality of evidence was downgraded by one for each outcome. The level of evidence and the reasons for the upgrades and downgrades are shown in Table 6. The ratings for the quality of evidence for the overall outcomes ranged from moderate to very low because of performance bias and obvious heterogeneities. The ratings for evidence quality were as follows: moderate for ROM; low for the VAS, Lysholm scores, and knee circumferences; and very low for the IKDC subjective and HSS scores.

There are several limitations to this review. First, owing to the inconsistency of the TCM intervention types, duration, and evaluation time points, high heterogeneity was observed during the meta-analysis, although part of it was offset by the subgroup analysis. For higher quality meta-analyses, RCTs that adopted standardized TCM treatments for ACLR patients should be published in the future. Second, several studies with high risks of bias were included in this review. Well-designed RCTs with high-quality methodologies should be conducted in the future. Third, most of the included studies were conducted in China [13–30] except for one study from Spain [31]. Since TCM treatments are commonly performed in China, their cultural customs may act as another source of bias in this review. Therefore, more geographically diverse RCTs are encouraged in the future.

In summary, TCM can relieve pain, enhance the mobility of the knee joint, reduce edema after surgery, and improve several symptoms and the function of the knee joint after ACLR. In addition, the TCM treatments that were more diverse and had longer treatment durations and evaluation time points were more effective at improving the Lysholm scores and IKDC subjective scores after ACLR. Despite these promising results, the strength of evidence is weak and no definite conclusions can be drawn, given that the overall methodological quality of the studies was relatively low and the heterogeneity was not entirely resolved by the subgroup analysis. Nevertheless, this paper is the first systematic review to evaluate the efficacy of postoperative care using TCM after ACLR and will be a useful cornerstone for future research on TCM for postoperative care after various musculoskeletal surgeries.

## 5. Conclusions

TCM can be combined with conventional rehabilitation to reduce postoperative pain and knee edema as well as increase the mobility and function of the knee joint after ACLR. However, this recommendation should be carefully applied in clinical practice because of the relatively low overall quality of the included RCTs. RCTs with high methodological quality and more standardized TCM treatments should be conducted in the future.

## Figures and Tables

**Table 1:** Characteristics of the included studies.

First author	Sample size (M/F)	Mean age (*m* ± *s*)		POD (*m* ± *s*)		Onset (*m* ± *s*)		
		E	C	E	C	E	C	
Chen 2016	100 (61/39)	32.5 ± 2.5		—	—	3 d∼3 y		
Hu 2018	80 (64/16)	29.9 ± 4.6	28.3 ± 9.6	—	—	7 h∼7 d		
Huang 2018	76 (56/20)	32.32 ± 6.34	30.95 ± 5.73	4.12 ± 0.56 m	3.89 ± 0.31 m			
Jorge 2017	44 (28/16)	31.4 ± 8.3	34.4 ± 8.6	15.6 ± 1.5 d	15.5 ± 2.0 d			
Li 2017	60 (31/29)	25.2 ± 4.3	24.9 ± 3.2	—	—	7.4 ± 2.6 d	7.2 ± 3.1 d	
Liang 2006	32 (19/13)	29.0 ± 3.0	28.0 ± 11.0	—	—	87.6 ± 121.8 d	96.9 ± 117.7 d	
Liang 2010	76 (45/31)	-	-	—	—			
Tang 2017	60 (37/23)	28.8 ± 5.87	29.27 ± 6.58	—	—	4.67 ± 0.89 d	4.82 ± 0.65 d	
Wang 2017	60 (37/23)	37.80 ± 6.66	37.13 ± 6.47	—	—	8.55 ± 2.33 d	8.86 ± 1.91 d	
Wang et al. 2019	46 (13/33)	34.0	34.3	—	—			
Wang 2020	160 (95/65)	25.57 ± 7.55	25.11 ± 7.29	—	—	2.02 ± 0.93 d	2.11 ± 0.89 d	
Wang 2019	146 (99/47)	34.8	32.8	—	—	2–12 w		
Wei 2016	40 (20/20)	26.75 ± 6.20	29.85 ± 5.32	—	—			
Wu 2009	25 (17/8)	28.6	—	—	4h–11w			
Xiong et al. 2011	27 (17/10)	28.6	—	—	4h–11w			
Xu 2017	47 (27/20)	33.08 ± 9.85	33.56 ± 8.89	—	—			
Yang 2017	70 (59/11)	29.14 ± 7.62	29.23 ± 8.48	—	—	101.65 ± 33.25 d	105.29 ± 25.81 d	
Yi 2018	80 (53/27)	36.27 ± 12.85	35.85 ± 12.23	—	—	2.82 ± 0.81 m	2.95 ± 0.75 m	
Zhong 2019	60 (47/13)	29.00 ± 4.37	27.80 ± 4.84	—	—			

First author	Experimental group			Control group		Outcomes	Evaluation time points	Adverse events
	Intervention type	Duration	n	Intervention type	n			
Chen 2016	MA + EA + HM + FW + basic rehabilitation	54 d	50	Basic rehabilitation	50	VAS Lysholm score	3 m (POD3 m)	Not presented
Hu 2018	MA + EA + FW + MM + basic rehabilitation	4 w	40	Basic rehabilitation	40	VAS< ROM Lysholm score Knee circumference	4 w (POD4 w)	Not presented
Huang 2018	MA + WN + basic rehabilitation	4 w	38	Basic rehabilitation	38	VAS ROM Lysholm score	2 m (POD4 m–6 m)	Not presented
Jorge 2017	MA + basic rehabilitation	1 d	22	Basic rehabilitation	25	VAS ROM	1 d, 1 w, 5 w (POD6 w–8 w)	Presented
Li 2017	HM + FW + basic rehabilitation	4 w	30	Basic rehabilitation	30	Lysholm score Knee circumference	2 w, 4 w (POD4 w)	Not presented
Liang 2006	EA + HM + FW + MM + basic rehabilitation	46 d	16	Basic rehabilitation	16	VAS ROM Lysholm score IKDC-2000 Joint effusion	3 d, 1 w, 2 w, 3 m (POD3 m)	Not presented
Liang 2010	EA + HM + FW + MM + basic rehabilitation	4 w	38	Basic rehabilitation	38	VAS ROM Lysholm score IKDC-2000 HSS score Joint effusion	3 m, 6 m (POD6 m)	Presented
Tang 2017	MA + HM + FW + MM + basic rehabilitation	12 w	30	Basic rehabilitation	30	Lysholm score	3 m, 6 m, 12 m (POD12 m)	Not presented
Wang 2017	MA + basic rehabilitation	13 d	30	Basic rehabilitation	30	VAS ROM Knee circumference	2 d, 4 d, 6 d, 8 d, 10 d, 12 d, 2 w (POD2 w)	Not presented
Wang et al 2019	HM + basic rehabilitation	4 w	23	Basic rehabilitation	23	Lysholm score	2 w, 4 w, 6 w (POD6 w)	Not presented
Wang 2020	MA + basic rehabilitation	—	80	Basic rehabilitation	80	HSS score	4 m (POD4 m)	Not presented
Wang 2019	HM + basic rehabilitation	12 w	76	Basic rehabilitation	76	Lysholm score IKDC-2000	3 m, 6 m, 12 m (POD12 m)	Presented
Wei 2016	MA + EA + basic rehabilitation	4 d	20	Basic rehabilitation	20	VAS ROM	3 h, 6 h, 12 h, 1 d, 2 d, 3 d (POD3 d)	Presented
Wu 2009	HM + FW + MM + basic rehabilitation	5 w	13	Basic rehabilitation	12	VAS ROM Lysholm score IKDC-2000 Joint effusion	2 w, 4 w, 3 m (POD3 m)	Not presented
Xiong et al. 2011	HM + FW + MM + basic rehabilitation	7 w	15	Basic rehabilitation	12	VAS ROM Lysholm score IKDC-2000 Knee circumference	1 w, 2 w, 4 w, 3 m (POD3 m)	Not presented
Xu 2017	EA + HM + MM + basic rehabilitation	More than 4 w	24	Basic rehabilitation	23	VAS ROM Lysholm score	2 w, 1 m, 3 m (POD3 m)	Not presented
Yang 2017	HM + basic rehabilitation	1 y	35	Basic rehabilitation	35	Lysholm score IKDC-2000	6 m, 12 m (POD12 m)	Not presented
Yi 2018	MA + EA + basic rehabilitation	12 w	40	Basic rehabilitation	40	VAS HSS score	1 m, 3 m, 6 m (POD6 m)	Not presented
Zhong 2019	MA + basic rehabilitation	4 w	30	Basic rehabilitation	30	VAS Lysholm score Joint effusion	1 d, 5 d, 12 d, 1 m, 3 m (POD3 m)	Not presented

M: male; F: female; E: experimental group; C: control group; MA: manual acupuncture; WN: warm needling; EA: electroacupuncture; HM: herbal medicine; FW: fuming-washing therapy; MM: massage along meridian; VAS: visual analogue scale; ROM: range of motion; IKDC-2000: International Knee Documentation Committee 2000; WOMAC: Western Ontario and McMaster Universities Osteoarthritis Index; HSS: Hospital for Special Surgery; POD: postoperative date.

**Table 2:** Details of acupuncture treatment.

First author	Acupuncture rationale	Details of needling		
		Type of acupuncture	Acupuncture points	Needling duration (ES frequency)
Chen 2016	TCM	MA	GB31, GB34, ST36, ST40	15 min
		EA	BL57, GB31, GB34, SP6, SP10, ST32, ST36, ST40	15 min (80 Hz)

Hu 2018	TCM	MA	BL57, GB34, SP9, SP10, ST34, ST35, ST36	30 min
		EA	BL57, GB34, SP9, SP10, ST34, ST35, ST36	30 min (n.r.)

Huang 2018	TCM	MA	LR3, SP9, SP10, ST32, ST34, ST35, ST36, EX-LE4	30 min
Jorge 2017	Western medical	MA	Vastus medialis TrP	1-2 min
Liang 2006	TCM	EA	GB34, SP9, ST36	n.r. (80 Hz)
Liang 2010	TCM	EA	n.r.	n.r. (n.r.)
Tang 2017	TCM	MA	BL39, BL40, BL57, GB33, GB34, LR7, SP6, SP10, ST34, ST35, ST36	n.r.
Wang 2017	TCM	MA	SP6, SP9, SP10, ST32, ST34, ST36	30 min
Wang 2020	TCM	MA	GB34, GV20, KI3, LI4, LR3, SP6, SP9, SP10, ST34, EX-LE2, zuyundongqu	30 min
Wei 2016	TCM	MA	LR6	30 min
		EA	LR6	30 min (100–1000 Hz)

Xu 2017	TCM	EA	BL57, GB34, SP6, SP9, SP10, ST36, ST40	n.r.
Yi 2018	TCM	MA	BL39, BL40, BL57, GB33, GB34, LR7, SP6, SP9, SP10, ST34, ST35, ST36	30 min
		EA	BL39, BL40, BL57, GB33, GB34, LR7, SP6, SP9, SP10, ST34, ST35, ST36	30 min (n.r.)

Zhong 2019	TCM	MA	GB34, SP6, SP9, SP10, ST32, ST34, ST36	20 min

TCM: traditional Chinese medicine; MA: manual acupuncture; EA: electroacupuncture; ES: electrical stimulation; n.r: not reported.

**Table 3:** Frequently used acupuncture points.

Number of times used	9	8	7	5	4
Acupuncture point	SP10, ST36	GB34, SP9	SP6, ST34	BL57	ST32, ST35

**Table 4:** Details of herbal medicine treatment.

First author	Dosage form	Administration duration and frequency	Herbal medicine name	Basic components
Chen 2016	Decoction	3 w, tid	1. Xishangyihao-fang	1. Achyranthis Radix, Coptidis Rhizoma, Curcumae Longae Radix, Rhei Rhizoma Preparata Cum Vinum
			2. Xishangerhao-fang	2. Angelicae Gigantis Radix, Bletillae Rhizoma, Cyperi Rhizoma, Coptidis Rhizoma, Persicae Semen
			3. Xishangsanhao-fang	3. Astragali Radix, Chaenomelis Fructus, Cibotii Rhizoma, Codonopsis Pilosulae Radix, Homalomenae Rhizoma

Li 2017	Tablet preparation	4 w, tid	1. Guxiyi decoction	1. Achyranthis Radix, Angelicae Gigantis Radix, Araliae Continentalis Radix, Aucklandiae Radix, Cnidi Fructus, Cnidii Rhizoma, Coicis Semen, Drynariae Rhizoma, Glycyrrhizae Radix Et Rhizoma, Eupolyphaga, Mori Ramulus, Moutan Cortex Radicis, Myrrha, Olibanum, Notoginseng Radix, Paeoniae Radix, Phryma Leptostachya, Syzygii Flos, Rehmanniae Radix Crudus, Salviae Miltiorrhizae Radix

Liang 2006	Decoction	4 w, tid	1. Xishangyihao-fang	1. Achyranthis Radix, Coptidis Rhizoma, Curcumae Longae Radix, Rhei Rhizoma Preparata Cum Vinum, Scutellariae Radix, Taraxaci Herba
			2. Xishangerhao-fang	2. Achyranthis Radix, Akebiae Caulis, Angelicae Gigantis Radix, Bletillae Rhizoma, Coptidis Rhizoma, Cyperi Rhizoma, Dipsaci Radix, Paeoniae Radix, Persicae Semen
			3. Xishangsanhao-fang	3. Akebiae Caulis, Astragali Radix, Chaenomelis Fructus, Cibotii Rhizoma, Cistanchis Herba, Codonopsis Pilosulae Radix, Cuscutae Semen, Lumbricus, Homalomenae Rhizoma, Visci Herba Et Loranthi Ramulus

Liang 2010	Decoction	4 w, tid	1. Xishangyihao-fang	—
			2. Xishangerhao-fang	

Tang 2017	Decoction, powder preparation	6 w, tid	1. XiaoZhong ZhiTong mixture	—
			2. Sunshangsan	

Wang et al. 2019	Decoction	4 w, bid	1. Taohongsiwu-tang	1. Angelicae Gigantis Radix, Carthami Flos, Cnidii Rhizoma, Paeoniae Radix, Persicae Semen, Rehmanniae Radix Preparata

Wang 2019	Pill preparation	12 w, bid	1. Bujin-wan	1. Acanthopanax Root Bark, Achyranthis Radix, Angelicae Gigantis Radix, Aucklandiae Radix, Chaenomelis Fructus, Cistanchis Herba, Cnidi Fructus, Cuscutae Semen, Dioscoreae Rhizoma, Ginseng Radix, Olibanum, Poria Sclerotium, Rehmanniae Radix Preparata, Tribuli Fructus

Wu 2009	Decoction	30 d, bid-tid	1. Xishangyihao-fang	1. Achyranthis Radix, Coptidis Rhizoma, Curcumae Longae Radix, Scutellariae Radix, Taraxaci Herba
			2. Xishangerhao-fang	2. Achyranthis Radix, Akebiae Caulis, Angelicae Gigantis Radix, Bletillae Rhizoma, Carthami Flos, Coptidis Rhizoma, Cyperi Rhizoma, Dipsaci Radix, Paeoniae Radix, Persicae Semen
			3. Xishangsanhao-fang	3. Akebiae Caulis, Angelicae Gigantis Radix, Astragali Radix, Chaenomelis Fructus, Cibotii Rhizoma, Cistanchis Herba, Cuscutae Semen, Homalomenae Rhizoma, Lumbricus, Visci Herba Et Loranthi Ramulus

Xiong 2011	Decoction	30 d	1. Xishangyihao-fang	1. Achyranthis Radix, Coptidis Rhizoma, Curcumae Longae Radix, Scutellariae Radix, Taraxaci Herba
			2. Xishangerhao-fang	2. achyranthis radix, akebiae caulis, angelicae gigantis radix, bletillae rhizoma, carthami flos, coptidis rhizoma, cyperi rhizoma, dipsaci radix, paeoniae radix, persicae semen
			3. Xishangsanhao-fang	3. Akebiae Caulis, Angelicae Gigantis Radix, Astragali Radix, Chaenomelis Fructus, Cibotii Rhizoma, Cistanchis Herba, Cuscutae Semen, Homalomenae Rhizoma, Lumbricus, Visci Herba et Loranthi Ramulus

Xu 2017	Soluble granules, pill preparation	More than 4 w, tid	1. Huoxuezhitong-Jiaonang	1. Angelicae Gigantis Radix, Bomeolum, Eupolyphaga, Notoginseng Radix, Olibanum, Pyritum
			2. Jianbuhuqian-wan	2. Aconiti Lateralis Radix Preparata, Angelicae Gigantis Radix, Araliae Continentalis Radix, Atractylodes Macrocephala Koidzumi, Chaenomelis Fructus, Cuscutae Semen, Cynomorium Songaricum Ruprecht, Dipsaci Radix, Eucommiae Cortex, Gentianae Macrophyllae Radix, Ginseng Radix, Lycii Fructus, Osterici Radix, Paeoniae Radix, Poria Sclerotium, Psoraleae Semen, Rehmanniae Radix Preparata, Saposhnikoviae Radix, Testudinis Plastrum

Yang 2018	Pill preparation	12 m, bid	1. Liuwei dihuang pills	1. Alismatis Rhizoma, Corni Fructus, Dioscoreae Rhizoma, Moutan Cortex Radicis, Poria Sclerotium, Rehmanniae Radix Preparata

**Table 5:** Frequently used Chinese medicinal herbs.

Number of times used	8	6	5	4
Chinese medicinal herbs	Angelicae Gigantis Radix	Achyranthis Radix Chaenomelis Fructus Paeoniae Radix	Cuscutae Semen Persicae Semen	Astragali Radix Bletillae Rhizoma Cibotii Rhizoma Cistanchis Herba Coptidis Rhizoma Curcumae Longae Radix Cyperi Rhizoma Dipsaci Radix Homalomenae Rhizoma Rehmanniae Radix Preparata

**Table 6:** Summary of findings.

Outcomes	Anticipated absolute effects^*∗*^ (95% CI)		Relative effect (95% CI)	Number of participants (studies)	Certainty of the evidence (GRADE)	Comments
	Risk with CR	Risk with TCM + CR				
VAS Scale from: 0 to 10 follow-up: range 3 days to 6 months	The mean VAS ranged from 1.06 to 4.02 points.	MD 0.74 points lower (0.93 lower to 0.54 lower)	—	780 (13 RCTs)	⊕⊕○○ LOW a, b	Patients who received TCM after ACLR had lower VAS score with some uncertainty due to performance bias and considerable heterogeneity.
ROM Scale from: 0 to 180 follow-up: range 3 days to 6 months	—	SMD 1.19 SD higher (0.78 higher to 1.59 higher)	—	540 (10 RCTs)	⊕⊕⊕○ MODERATE b	Patients who received TCM after ACLR had better ROM with moderate certainty due to considerable heterogeneity.
Lysholm score Scale from: 0 to 100 follow-up: range 4 weeks to 1 years	The mean Lysholm score ranged from 49.2–91.79 points.	MD 5.62 points higher (3.93 higher to 7.32 higher)	—	914 (14 RCTs)	⊕⊕○○ LOW a, b	Patients who received TCM after ACLR had a better Lysholm score with some uncertainty due to performance bias and considerable heterogeneity.
IKDC subjective score Scale from: 0 to 100 follow-up: range 3 months to 12 months	The mean IKDC subjective score ranged from 49.13 to 90.5 points.	MD 3.4 points higher (0.61 lower to 7.41 higher)	—	376 (6 RCTs)	⊕○○○ VERY LOW a, b, c	There was no significant difference in the IKDC subjective score between the TCM and control groups with uncertainty due to performance bias, considerable heterogeneity, and low population size.
HSS score Scale from: 0 to 100 follow-up: range 4 months to 6 months	The mean HSS score ranged from 56.18 to 92.24 points.	MD 6.79 points higher (1.27 lower to 14.86 higher)	—	316 (3 RCTs)	⊕○○○ VERY LOW a, b, c	There was no significant difference in the HSS score between the TCM and control groups with uncertainty due to performance bias, considerable heterogeneity, and low population size.
Knee circumference follow-up: range 2 weeks to 3 months	—	SMD 1.72 SD lower (2.38 lower to 1.07 lower)	—	227 (4 RCTs)	⊕⊕○○ LOW b, c	Patients who received TCM after ACLR had lesser knee swelling with some uncertainty due to performance bias and considerable heterogeneity.
VAS Scale from: 0 to 10 follow-up: range 3 days to 6 months	The mean VAS ranged from 1.06 to 4.02 points.	MD 0.74 points lower (0.93 lower to 0.54 lower)	—	780 (13 RCTs)	⊕⊕○○ LOW a, b	Patients who received TCM after ACLR had a lower VAS score with some uncertainty due to performance bias and considerable heterogeneity.

^a^Unclear performance bias which may affect outcomes. ^b^Considerable heterogeneity was detected. However, most inconsistency was explained by differences in interventions, duration, and evaluation time points. ^c^Population size less than 400. CI: confidence interval; MD: mean difference; SMD: standardized mean difference; VAS: visual analogue scale; ROM: range of motion; IKDC: International knee documentation committee; HSS: hospital for special surgery; RCTs: randomized controlled trials; ACLR: anterior cruciate ligament reconstruction; TCM: traditional Chinese medicine.

## Data Availability

Data will be provided upon request to the corresponding author.

## Appendix

Search strategies for MEDLINE/PubMed databases are as follows (Access Date: 7 June 2020):

1. anterior cruciate ligament reconstruction [MeSH]
2. anterior cruciate ligament injuries [MeSH]
3. “anterior cruciate ligament repair” [tw]
4. “anterior cruciate ligament” [tw]
5. 1 OR 2 OR 3 OR 4
6. “anterior cruciate ligament” [tw]
7. “intra‐articular knee ligament” [tw]
8. 6 OR 7
9. injury OR rupture OR torn OR destruction OR trauma OR reconstruction OR repair
10. 8 AND 9
11. 5 OR 10
12. acupuncture therapy [MeSH]
13. acupuncture [MeSH]
14. acupuncture point [MeSH]
15. “acupuncture needle” [tw]
16. meridians [MeSH]
17. acupuncture^*∗*^ [tw]
18. needle^*∗*^ [tw]
19. acupoint^*∗*^ [tw]
20. electroacupuncture [MeSH]
21. electroacupuncture [tw]
22. pharmacoacupunctur^*∗*^ [tw]
23. pharmacoacupunctur^*∗*^ [tw]
24. “acupoint injection” [tw]
25. “auricular acupunctur^*∗*^” [tw]
26. “ear acupunctur^*∗*^” [tw]
27. “auricular needl^*∗*^” [tw]
28. “ear needl^*∗*^” [tw]
29. “fire acupunctur^*∗*^” [tw])
30. “scalp acupuncture^*∗*^” [tw]
31. “warm acupunctur^*∗*^” [tw]
32. “warm needl^*∗*^” [tw]
33. 12 OR 13 OR 14 OR 15 OR 16 OR 17 OR 18 OR 19 OR 20 OR 21 OR 22 OR 23 OR 24 OR 25 OR 26 OR 27 OR 28 OR 29 OR 30 OR 31 OR 32
34. Medicine, Chinese Traditional [MeSH]
35. Chinese herbal medicine [tw]
36. Chinese medicine [tw]
37. Chinese herbal drug [tw]
38. traditional herbal medicine [tw]
39. herbal medicine [tw]
40. decoction [tw]
41. tang [tw]
42. ^*∗*^tang [tw]
43. formula [tw]
44. 34 OR 35 OR 36 OR 37 OR 38 OR 39 OR 40 OR 41 OR 42 OR 43
45. 33 OR 44
46. “randomized controlled trial” [Publication Type]
47. “randomized controlled trials as topic” [MeSH]
48. “random allocation” [MeSH]
49. “double-blind method” [MeSH]
50. “single-blind method” [MeSH]
51. placebo [MeSH]
52. random^*∗*^ [tw]
53. rct [tw]
54. rct's [tw]
55. rcts [tw]
56. placebo^*∗*^ [tw]
57. 46 OR 47 OR 48 OR 49 OR 50 OR 51 OR 52 OR 53 OR 54 OR 55 OR 56
58. 11 AND 45 AND 57
59. Search Results: 14

Search strategies for EMBASE database are as follows (Access Date: 7 June 2020):

(31) (13) AND (20) AND (30)
(30) (21) OR (22) OR (23) OR (24) OR (25) OR (26) OR (27) OR (28) OR (29)
(29) placebo^*∗*^: ab, ti
(28) rcts: ab, ti
(27) rct: ab, ti
(26) random^*∗*^: ab, ti
(25) “placebo”/exp
(24) “single blind procedure”/exp
(23) “double blind procedure”/exp
(22) “randomization”/exp
(21) “randomized controlled trial (topic)”/exp
(20) (14) OR (15) OR (16) OR (17) OR (18) OR (19)
(19) “formula”:ab, ti
(18) “-tang”:ab, ti
(17) “decoction”:ab, ti
(16) “herbal medicine”/exp
(15) “Chinese medicine equipment”/exp
(14) “Chinese medicine”/exp
(13) (5) OR (12)
(12) (8) AND (11)
(11) (9) OR (10)
(10) reconstruction:ab, ti OR repair:ab, ti
(9) injury:ab, ti OR rupture:ab, ti OR torn:ab, ti OR destruction:ab, ti OR trauma:ab, ti
(8) (6) OR (7)
(7) “intra‐articular knee ligament”: ab, ti
(6) “anterior cruciate ligament”: ab, ti
(5) (1) OR (2) OR (3) OR (4)
(4) “anterior cruciate ligament”: ab, ti
(3) “anterior cruciate ligament repair”: ab, ti
(2) “anterior cruciate ligament injury”/exp
(1) “anterior cruciate ligament reconstruction”/exp
  Search Results: 21

Search strategies for CENTRAL database are as follows (Access Date: 7 June 2020):

1. MeSH descriptor: [Anterior Cruciate Ligament Reconstruction] explode all trees
2. MeSH descriptor: [Anterior Cruciate Ligament Injuries] explode all trees
3. “Anterior Cruciate Ligament repair”
4. “Anterior Cruciate Ligament”
5. (1) OR (2) OR (3) OR (4)
6. “anterior cruciate ligament”
7. “intraarticular knee ligament”
8. (6) OR (7)
9. (injury OR rupture OR torn OR destruction OR trauma)
10. (reconstructions OR repair)
11. (9) OR (10)
12. (8) AND (11)
13. (5) OR (12)
14. MeSH descriptor: [Medicine, Chinese Traditional] explode all trees
15. MeSH descriptor: [Herbal Medicine] explode all trees
16. MeSH descriptor: [Drugs, Chinese Herbal] explode all trees
17. traditional herbal medicine
18. decoction
19. formula
20. ^*∗*^tang
21. (14) OR (15) OR (16) OR (17) OR (18) OR (19) OR (20)
22. MeSH descriptor: [Randomized Controlled Trial] explode all trees
23. MeSH descriptor: [Randomized Controlled Trials as Topic] explode all trees
24. MeSH descriptor: [Random Allocation] explode all trees
25. MeSH descriptor: [Double-Blind Method] explode all trees
26. MeSH descriptor: [Single-Blind Method] explode all trees
27. MeSH descriptor: [Placebos] explode all trees
28. random^*∗*^
29. rct
30. rct's
31. rcts
32. placebo^*∗*^
33. (22) OR (23) OR (24) OR (25) OR (26) OR (27) OR (28) OR (29) OR (30) OR (31) OR (32)
34. (13) AND (21) AND (33)
35. Search Results: 12

Search strategies for CNKI database are as follows (Access Date: 7 June 2020):

  Subject category: Medicine and Public Health
  Sub-database: Journal articles, Dissertations
  Search strategy:
  (SU = “前交叉韧带” + “前交叉韧带重建术”) AND (SU = “中医治疗” + “中医治疗结合” + “针灸” + “针刺” + “中药”)
  Search Results: 207

## Abbreviations

ACL: Anterior cruciate ligament
ACLR: Anterior cruciate ligament reconstruction
CGRP: Calcitonin gene-related peptide
CI: Confidence intervals
CNKI: Chinese National Knowledge Infrastructure
CPM: Continuous passive motion therapy
CR: Conventional rehabilitation
EA: Electroacupuncture
HSS: Hospital for special surgery
IKDC: International Knee Documentation Committee 2000
MDs: Mean differences
NO: Nitric oxide
NMES: Neuromuscular electrical stimulation
OR/IF: Open reduction and internal fixation
RCT: Randomized controlled trial
ROM: Range of motion
SMDs: Standardized mean differences
TCM: Traditional Chinese medicine
TKA: Total knee arthroplasty
TKR: Total knee replacement
TrPs: Trigger points
VAS: Visual analogue scale
WMDs: Weighted mean differences
WOMAC: Western Ontario and Mcmaster Universities Osteoarthritis Index
JMAS: Japan Medical Abstracts Society.
